# P-tau subgroups in AD relate to distinct amyloid production and synaptic integrity profiles

**DOI:** 10.1186/s13195-022-01038-z

**Published:** 2022-07-15

**Authors:** Kirsten E. J. Wesenhagen, Betty M. Tijms, Lynn Boonkamp, Patty L. Hoede, Julie Goossens, Nele Dewit, Philip Scheltens, Eugeen Vanmechelen, Pieter Jelle Visser, Charlotte E. Teunissen

**Affiliations:** 1 Alzheimer Center Amsterdam, Neurology, Vrije Universiteit Amsterdam, Amsterdam UMC location VUmc, Amsterdam, The Netherlands; 2 Amsterdam Neuroscience, Neurodegeneration, Amsterdam, The Netherlands; 3 Neurochemistry Lab, Department of Clinical Chemistry, Amsterdam Neuroscience, Amsterdam UMC, Vrije Universiteit, Amsterdam, The Netherlands; 4 ADx NeuroSciences, Ghent, Belgium; 5 Alzheimer Center Limburg, School for Mental Health and Neuroscience, Maastricht University, Maastricht, The Netherlands; 6 Department of Neurobiology, Care Sciences and Society, Division of Neurogeriatrics, Karolinska Institutet, Stockholm, Sweden

**Keywords:** Alzheimer’s disease, Cerebrospinal fluid, p-tau, Biological heterogeneity

## Abstract

**Background:**

We previously identified four Alzheimer’s disease (AD) subgroups with increasingly higher cerebrospinal fluid (CSF) levels of tau phosphorylated at threonine 181 (p-tau). These subgroups included individuals across the cognitive spectrum, suggesting p-tau subgroups could reflect distinct biological changes in AD, rather than disease severity. Therefore, in the current study, we further investigated which potential processes may be related with p-tau subgroups, by comparing individuals on CSF markers for presynaptic structure [vesicle-associated membrane protein 2 (VAMP2)], postsynaptic structure [neurogranin (NRGN)], axonal damage [neurofilament light (NfL)], and amyloid production [beta-secretase 1 (BACE1) and amyloid-beta 1–40 (Aβ40)].

**Methods:**

We selected 348 amyloid-positive (A+) individuals (53 preclinical, 102 prodromal, 193 AD dementia) and 112 amyloid-negative (A−) cognitively normal (CN) individuals from the Amsterdam Dementia Cohort (ADC). Individuals were labeled according to their p-tau subgroup (subgroup 1: p-tau ≤ 56 pg/ml; subgroup 2: 57–96 pg/ml; subgroup 3: 97–159 pg/ml; subgroup 4: > 159 pg/ml). CSF protein levels were measured with ELISA (NRGN, BACE1, Aβ40, NfL) or single-molecule array (Simoa) (VAMP2). We tested whether protein levels differed between the p-tau subgroups within A+ individuals with linear models corrected for age and sex and whether disease stage influenced these relationships.

**Results:**

Among A+ individuals, higher p-tau subgroups showed a higher percentage of AD dementia [subgroup 1: *n* = 41/94 (44%); subgroup 2: *n* = 81/147 (55%); subgroup 3: *n* = 59/89 (66%); subgroup 4: *n* = 7/11 (64%)]. Relative to controls, subgroup 1 showed reduced CSF levels of BACE1, Aβ40, and VAMP2 and higher levels of NfL. Subgroups 2 to 4 showed gradually increased CSF levels of all measured proteins, either across the first three (NfL and Aβ40) or across all subgroups (VAMP2, NRGN, BACE1). The associations did not depend on the clinical stage (interaction *p*-values ranging between 0.19 and 0.87).

**Conclusions:**

The results suggest that biological heterogeneity in p-tau levels in AD is related to amyloid metabolism and synaptic integrity independent of clinical stage. Biomarkers reflecting amyloid metabolism and synaptic integrity may be useful outcome measures in clinical trials targeting tau pathology.

## Background

One of the main pathological hallmarks of Alzheimer’s disease (AD) is the aggregation of hyperphosphorylated tau proteins into tangles in the brain. The burden of tau pathology is associated with cognitive decline [1]. Tau protein concentrations can be measured in cerebrospinal fluid (CSF) as total tau (t-tau) or tau phosphorylated at threonine 181 (p-tau). While both t-tau and p-tau are used as core AD biomarkers in the research framework’s definition of AD [2], p-tau levels are considered to be more specific for neurofibrillary tangles [2] and to differentiate AD from other neurodegenerative diseases with higher specificity [3]. Usually, a single cutpoint is used to separate normal and abnormal p-tau values [2]. However, we have recently identified four subgroups with increasingly high CSF p-tau values [4]. The subgroups included individuals across the clinical spectrum, and most individuals remained in the same p-tau subgroup over time, suggesting that p-tau subgroups may reflect different underlying pathophysiological processes.

Previous studies have found that in addition to amyloid and tau pathology, multiple additional biological processes are involved in AD, including, e.g., amyloid homeostatic changes [5], synaptic dysfunction [6–8], and axonal damage [9]. Changes in these processes can be investigated by measuring the levels of biomarkers in CSF. Amyloid production is reflected, among others, by levels of beta-site amyloid precursor protein cleaving enzyme 1 (BACE1). BACE1 is part of the amyloidogenic pathway producing amyloid-beta 1–42 (Aβ42) [10]. In previous studies, BACE1 levels in CSF were increased in AD-type dementia relative to controls [11] and correlated with t-tau and p-tau levels [12, 13]. Amyloid-beta 1–40 (Aβ40) is another biomarker reflecting amyloid production and is considered to reflect overall production of amyloid-beta species [14]. Aβ40 levels were shown to correlate with p-tau levels in AD [10], but results conflicted whether Aβ40 levels are increased in AD-type dementia relative to controls [9, 10]. Synaptic integrity is reflected by biomarkers such as vesicle-associated membrane protein 2 (VAMP2) and neurogranin (NRGN). VAMP2 is a presynaptic protein involved in neurotransmitter release [8, 15], and a previous study showed its levels correlate with t-tau levels and are increased in early stages of AD [16]. NRGN is a postsynaptic protein involved in synaptic signaling and remodeling (see review [17]), and in previous studies, NRGN levels correlated with t-tau and p-tau levels [8, 18–22] and were increased in (early) AD relative to controls [20, 21, 23]. Finally, levels of the axonal structural protein neurofilament light (NfL) increase in CSF with axonal damage [24] and are correlated with t-tau and p-tau levels [25]. Levels of NfL are increased in prodromal AD and AD-type dementia [19], but also in other neurodegenerative diseases [19, 24], and NfL is therefore considered a non-specific marker of neuronal damage.

Since previous studies showed that biomarkers of amyloid metabolism, synaptic integrity, and axonal damage correlate with CSF tau values, we hypothesized that p-tau subgroups in individuals with AD may differ in these processes. We investigated in individuals with AD the relationships of p-tau subgroups with proteins reflecting amyloid production (BACE1, Aβ40), synaptic function (VAMP2, NRGN), and axonal damage (NfL) and tested if such associations depended on clinical stage. Finally, since an increase in tau is observed in normal aging as well [26], we tested if protein levels were similarly associated with p-tau in cognitively normal (CN) individuals with normal amyloid.

## Methods

### Study cohort and selection criteria

The present analyses were conducted as part of the redefining AD study, which aims to explain biological heterogeneity in AD and is described in this paper for the first time. We selected individuals from the Amsterdam Dementia Cohort (ADC) [27] when they had normal cognition and normal CSF amyloid or when they had abnormal amyloid (A+) across the clinical AD spectrum (i.e., normal cognition (preclinical AD), mild cognitive impairment (prodromal AD), and AD-type dementia). Individuals further needed to have available CSF stored in our biobank. This resulted in a selection of 453 individuals. The study participants visited our memory clinic between September 2004 and March 2018.

As described in more detail elsewhere [27], most individuals received a standardized neuropsychological test battery with at least one test per cognitive domain and completed the Mini-Mental State Examination (MMSE) [28]. In addition, participants underwent physical and neurological investigation, laboratory tests, and imaging [electroencephalography (EEG) and magnetic resonance imaging (MRI)]. The diagnosis was made by a multidisciplinary team blinded to the patients’ CSF Aβ42, t-tau, and p-tau results. For MCI, Petersen’s criteria [29] were used until the start of 2012, after which the criteria were used of the National Institute on Aging and Alzheimer’s Association (NIA-AA) [30]. For AD dementia, the criteria were used of the National Institute of Neurological and Communicative Disorders and Stroke-Alzheimer’s Disease and Related Disorders Association (NINCDS-ADRDA) [31]. Individuals who did not meet the criteria for MCI, AD, other dementias, or any psychiatric or neurologic disease were considered CN. All study participants gave written informed consent for the use of their clinical data and CSF for research purposes. The ADC study was approved by the ethical review board of the VU University Medical Center.

### Collection of CSF by lumbar puncture

Lumbar puncture was performed using a 25-gauge needle and a syringe, and CSF was collected in 10-mL polypropylene tubes (Sarstedt, Nümbrecht, Germany) [32, 33]. The CSF was centrifuged at 1800*g* at 4 °C for 10 min within 2 h of the lumbar puncture, transferred to new polypropylene tubes, and stored at − 20 °C for routine analysis of core AD biomarkers, or stored in the biobank at − 80 °C.

### Amyloid, t-tau, and p-tau measurements

Levels of amyloid, t-tau, and p-tau in CSF were measured with Innotest on a routine basis [34]. We used drift-corrected historical amyloid values, because values in ADC showed an upward drift across time [35], and used a cutpoint of 813 pg/ml to dichotomize amyloid status. We defined p-tau subgroups using previously derived cutpoints [4]: subgroup 1 (p-tau ≤ 56 pg/ml), subgroup 2 (p-tau 57–96 pg/ml), subgroup 3 (p-tau 97–159 pg/ml), and subgroup 4 (p-tau > 159 pg/ml). We additionally determined dichotomous t-tau and p-tau status based on the lowest of our previously derived cutpoints [4], with levels considered abnormal at p-tau > 56 pg/ml and t-tau > 349 pg/ml.

### Protein measurements

Levels of BACE1, Aβ40, and NRGN in CSF were measured with ELISA assays (EUROIMMUN, Germany) according to the manufacturer’s instructions. Levels of VAMP2 were measured with single-molecule array (Simoa) technology (Quanterix Corp, Billerica, USA) using a prototype assay developed by ADx NeuroSciences (Belgium), and levels of NfL were measured with an ELISA assay developed by ADx NeuroSciences (Belgium). The novel NfL assay correlated well (Spearman *R* = 0.952) with the widely used NF-Light ELISA (UmanDiagnostics, Sweden) (Das et al., manuscript submitted). For VAMP2, we excluded 21 measurements with a coefficient of variation (CV) of 20 or higher, and 22 samples for which one of the two measurements failed. For NfL, we excluded 3 measurements which were lower than the limit of detection. NRGN, BACE1, and Aβ40 data had no missing values.

### Determination of *APOE* genotypes

*APOE* genotypes were determined in 10 mL EDTA blood from which DNA was isolated with the QIAamp DNA blood isolation kit (Qiagen), followed by genotyping with the Light Cycler *APOE* mutation detection kit (Roche Diagnostics GmbH, Germany).

### Statistics

Group differences between p-tau subgroups in demographic variables were tested with ANOVA, Kruskal-Wallis rank sum test, or chi-square tests, followed by post hoc subgroup comparisons with *t*-tests, Wilcoxon rank sum tests, or chi-square tests as applicable. We next tested if p-tau subgroups showed different protein levels among individuals with AD, first based on raw protein levels. As raw protein levels were non-normally distributed, we tested if the protein levels differed depending on p-tau subgroups with Kruskal-Wallis rank sum tests, and used Wilcoxon rank sum tests to compare all p-tau subgroups. To further test the subgroup differences adjusting for age and sex with linear models, we first natural log-transformed and then *Z*-scored protein levels relative to controls (i.e., CN individuals with normal amyloid, t-tau, and p-tau). All models had as outcome protein level and included as predictors p-tau subgroup, age, and sex. All effect sizes (i.e., estimated protein levels per p-tau subgroup and differences between p-tau subgroups) were adjusted for the other predictors. In all models, biomarker normal controls were used as a reference. We first constructed linear models across all individuals to test whether the analyzed proteins differed between p-tau subgroups. Next, we constructed linear models that additionally included an interaction term between p-tau subgroup and cognitive stage. We tested if p-tau subgroups showed an interaction with cognitive stage (considered at interaction *p*-value < 0.1) and estimated marginalized mean protein levels in each cognitive stage. Finally, we labeled CN amyloid-negative individuals as belonging to p-tau subgroups. A minority of these individuals had abnormal p-tau levels (p-tau subgroups 2 or 3) and could therefore be considered to have suspected non-Alzheimer’s disease pathophysiology (SNAP). We performed exploratory analyses if p-tau subgroups show differences in protein levels in CN amyloid-negative individuals by also constructing linear models in this group. Post hoc group comparisons were *p*-value adjusted with the false discovery rate (FDR) method (for Wilcoxon rank sum tests used on raw protein levels) or with the Sidak method (for contrasts between p-tau subgroups in linear models) and considered significant at an adjusted *p*-value < 0.05. All statistical analyses were performed in R version 3.6.1 “Action of the Toes,” and estimated marginal means were computed with the R package “emmeans” v1.4.

## Results

We included a total of 453 individuals, of whom 112 were amyloid-negative CN individuals (98 controls and 14 individuals with t-tau or p-tau levels indicative of SNAP) and of whom 341 were A+ across different clinical stages (preclinical AD (*n* = 51), prodromal AD (*n* = 102), AD dementia (*n* = 188); full demographic characterization in Table 1). Compared to amyloid-negative individuals, A+ individuals showed a higher proportion of *APOE* ε4 carriers and were on average older, with the oldest average age in prodromal AD.

**Table 1:** Demographics of study participants

	Amyloid-negative CN	Preclinical AD	Prodromal AD	AD dementia
*n*	112	51	102	188
Age in years, mean ± sd	58.6 ± 7.8^abc^	63.7 ± 7.9^ad^	66.9 ± 7.7^bdf^	65 ± 7.2^cf^
Sex, female (%)	38 (34%)	24 (47%)	35 (34%)	90 (48%)
*APOE* ε4 carriership (%)	34 (30%)^abc^	30 (59%)^a^	71 (70%)^b^	115 (61%)^c^
MMSE, mean ± sd	28.4 ± 1.4^bc^	28 ± 1.4^de^	26.4 ± 2^bdf^	21 ± 4.4^cf^
Amyloid, pg/ml, mean ± sd	1144 ± 167^abc^	651 ± 109^ae^	625 ± 100^bf^	593 ± 100^cf^
T-tau, pg/ml, mean ± sd	227 ± 85^abc^	500 ± 307^ae^	554 ± 319^bf^	717 ± 398^cf^
P-tau, pg/ml, mean ± sd	40.3 ± 14^abc^	72.1 ± 39^ae^	75.4 ± 34^bf^	86.2 ± 36^cf^
T-tau abnormal (%)	8 (7.1%)^abc^	31 (61%)^ae^	73 (72%)^b^	152 (81%)^c^
P-tau abnormal (%)	9 (8%)^abc^	30 (59%)^ae^	70 (69%)^b^	147 (78%)^c^

Abnormal t-tau and p-tau status were based on previously derived cutoffs of 349 and 56 pg/ml (further details are in the “Methods” section). Differences in demographic variables between the diagnostic groups were tested with ANOVA, Wilcoxon rank sum test, or chi-square tests, followed by post hoc *t* tests, Wilcoxon rank sum tests, or chi-square tests as appropriate. *p*-values for the post hoc tests were FDR-adjusted to account for the multiple comparisons between the diagnostic groups

*CN*, cognitively normal

^a–f^Groups differed at *p*-value < 0.05

^a^Amyloid-negative CN vs preclinical AD

^b^Amyloid-negative CN vs prodromal AD

^c^Amyloid-negative CN vs AD dementia

^d^Preclinical AD vs prodromal AD

^e^Preclinical AD vs AD dementia

^f^Prodromal AD vs AD dementia

### Demographic comparisons of p-tau subgroups

We used our previously identified cutpoints to define four p-tau subgroups in our data. In total, of all A+ individuals across the AD clinical spectrum, 94 individuals fell in subgroup 1 (p-tau ≤ 56 pg/ml), 147 individuals in subgroup 2 (p-tau 57–96 pg/ml), 89 individuals in subgroup 3 (p-tau 97–159 pg/ml), and 11 individuals in subgroup 4 (p-tau > 159 pg/ml). A full demographic and biological characterization of p-tau subgroups is provided in Table 2. Relative to the first p-tau subgroup, the third p-tau subgroup showed an older age. P-tau subgroup 3 showed a higher proportion of women compared to subgroups 1–2. Higher p-tau subgroups tended to show worse MMSE scores in a stepwise manner. Overall, the relative proportion of preclinical and prodromal AD was similar for the p-tau subgroups, and the proportion of AD dementia was higher in subgroups 2–4 than in subgroup 1.

**Table 2:** Differences between p-tau subgroups in demographic variables and protein levels in CSF. Raw levels of proteins were compared between p-tau subgroups

	Characteristics per p-tau subgroup						*p*-values of p-tau subgroup comparisons									
	Controls (*n* = 98)	Subgroup 1: p-tau ≤ 56 pg/ml (*n* = 94)	Subgroup 2: p-tau 57–96 pg/ml (*n* = 147)	Subgroup 3: p-tau 97–159 pg/ml (*n* = 89)	Subgroup 4: p-tau > 159 pg/ml (*n* = 11)	*p*-value of group difference	Control-subgroup 1	Control-subgroup 2	Control-subgroup 3	Control-subgroup 4	Subgroup 1–2	Subgroup 1–3	Subgroup 1–4	Subgroup 2–3	Subgroup 2–4	Subgroup 3–4
Preclinical AD, *n* (percentage^a^)	0 (0%)	21 (22%)	20 (14%)	9 (10%)	1 (9.1%)	**7.2E−05**	n.t.	n.t.	n.t.	n.t.	1.000	1.000	1.000	1.000	1.000	1.000
Prodromal AD, *n* (percentage^a^)	0 (0%)	32 (34%)	46 (31%)	21 (24%)	3 (27%)	**2.4E−08**	n.t.	n.t.	n.t.	n.t.	**1.4E−02**	0.524	0.931	0.524	1.000	1.000
AD dementia, *n* (percentage^a^)	0 (0%)	41 (44%)	81 (55%)	59 (66%)	7 (64%)	**1.1E−21**	n.t.	n.t.	n.t.	n.t.	**0.0E+00**	**0.0E+00**	**8.9E−03**	0.178	0.977	1.000
Age in years, mean ± sd	58.2 ± 7.9	63.6 ± 7.8	65.5 ± 6.9	66.6 ± 7.8	68.5 ± 8.6	**6.0E−13**	**1.3E−05**	**2.0E−10**	**3.4E−10**	**7.4E−04**	0.119	**1.5E−02**	0.080	0.179	0.208	0.581
Sex, female (%)	32 (33%)	33 (35%)	56 (38%)	52 (58%)	8 (73%)	**4.2E−04**	0.836	0.661	**6.9E−03**	0.056	0.822	**1.2E−02**	0.073	**1.2E−02**	0.088	0.696
*APOE* ε4 carriership (%)	28 (29%)	62 (66%)	93 (63%)	54 (61%)	7 (64%)	**1.2E−07**	**0.0E+00**	**0.0E+00**	**1.0E−04**	0.108	1.000	1.000	1.000	1.000	1.000	1.000
MMSE score, mean ± sd	28.5 ± 1.4	24.6 ± 4.7	23.9 ± 4.2	22.4 ± 4.8	22.5 ± 4.3	**1.3E−28**	**1.8E−12**	**2.1E−22**	**3.7E−22**	**3.5E−06**	0.072	**6.1E−04**	0.086	**2.5E−02**	0.282	0.987
Amyloid in pg/ml, mean ± sd	1139 ± 161	616 ± 119	607 ± 99	615 ± 95	596 ± 95	**3.3E−48**	**2.7E−32**	**4.3E−39**	**1.4E−31**	**1.5E−07**	0.846	0.953	0.846	0.846	0.846	0.846
t-tau in pg/ml, mean ± sd	207 ± 59	286 ± 106	568 ± 154	982 ± 252	1732 ± 359	**2.4E−77**	**6.3E−10**	**1.4E−38**	**1.7E−31**	**7.2E−08**	**1.7E−31**	**5.7E−31**	**7.2E−08**	**6.0E−29**	**4.8E−08**	**7.6E−07**
p-tau in pg/ml, mean ± sd	36.7 ± 9.3	42.9 ± 11	75.4 ± 11	116 ± 16	195 ± 23	**4.0E−82**	**4.4E−06**	**4.2E−39**	**9.9E−32**	**7.8E−08**	**1.9E−38**	**3.2E−31**	**7.8E−08**	**2.2E−37**	**5.6E−08**	**7.8E−08**
Abnormal t-tau status, *n* (%)	0 (0%)	15 (16%)	141 (96%)	89 (100%)	11 (100%)	**2.2E−77**	n.t.	n.t.	n.t.	n.t.	**0.0E+00**	**0.0E+00**	**0.0E+00**	0.17	1	n.t.
Abnormal p-tau status, *n* (%)	0 (0%)	0 (0%)	147 (100%)	89 (100%)	11 (100%)	**1.0E−93**	n.t.	n.t.	n.t.	n.t.	n.t.	n.t.	n.t.	n.t.	n.t.	n.t.
BACE1 in ng/ml, median (range)	1.68 (.77–3.37)	1.51 (.63–2.47)	1.93 (1.04–3.48)	2.51 (1.54–3.93)	3.60 (2.61–4.34)	**2.1E−37**	**5.0E−03**	**2.0E−06**	**2.2E−18**	**1.3E−07**	**1.1E−13**	**2.2E−23**	**1.3E−07**	**7.0E−13**	**1.5E−07**	**7.4E−05**
Aβ40 in ng/ml, median (range)	6.09 (3.44–10.68)	5.02 (2.0–8.92)	6.71 (3.32–12.20)	8.69 (3.59–13.95)	9.40 (7.81–15.79)	**9.8E−34**	**2.8E−06**	**1.5E−02**	**8.4E−15**	**4.2E−06**	**3.3E−13**	**1.5E−23**	**2.2E−07**	**9.8E−13**	**1.1E−05**	0.069
NfL in pg/ml, median (range)	245 (113–1594)	389 (147–1770)	467 (169–2322)	630 (304–1441)	809 (413–1743)	**5.9E−37**	**5.5E−08**	**1.1E−20**	**3.4E−24**	**7.0E−07**	**4.5E−05**	**2.1E−14**	**3.1E−05**	**9.0E−09**	**4.1E−04**	0.107
VAMP2 in pg/ml, median (range)	131 (40–275)	106 (28–229)	163 (82–288)	237 (84–398)	382 (269–522)	**3.8E−41**	**2.9E−04**	**3.4E−07**	**6.9E−21**	**3.4E−07**	**8.3E−16**	**4.5E−24**	**3.4E−07**	**3.8E−15**	**3.2E−07**	**1.6E−05**
NRGN in pg/ml, median (range)	262 (73–498)	250 (56–501)	431 (188–752)	657 (353–1268)	1060 (754–1915)	**3.4E−63**	0.735	**9.3E−25**	**3.9E−30**	**8.1E−08**	**4.4E−25**	**9.2E−30**	**8.1E−08**	**4.3E−23**	**5.6E−08**	**1.2E−06**

Controls were cognitively normal and had normal CSF amyloid, t-tau, and p-tau. Abnormal t-tau and p-tau status were based on previously derived cutoffs of 349 and 56 pg/ml (further details are in the “Methods” section). Differences between p-tau subgroups in demographic variables were tested with ANOVA, Kruskal Wallis rank sum, or chi-square tests as appropriate, followed by pairwise *t* tests, Wilcoxon rank sum tests, or post hoc chi-square tests for subgroup comparisons as appropriate. Differences between p-tau subgroups in protein levels were all tested with the Kruskal Wallis rank sum test, followed by Wilcoxon rank sum tests. *p*-values were FDR-corrected for multiple tests between p-tau subgroups

*BACE1*, beta-site amyloid precursor protein cleaving enzyme 1; *Aβ40*, amyloid-beta 1–40; *NfL*, neurofilament light; *NRGN*, neurogranin; *VAMP2*, vesicle-associated membrane protein 2; *n.t.*, not tested

^a^Relative percentage in subgroup

### P-tau subgroups associated with amyloid production and synaptic biomarkers in AD

**Fig. 1:**
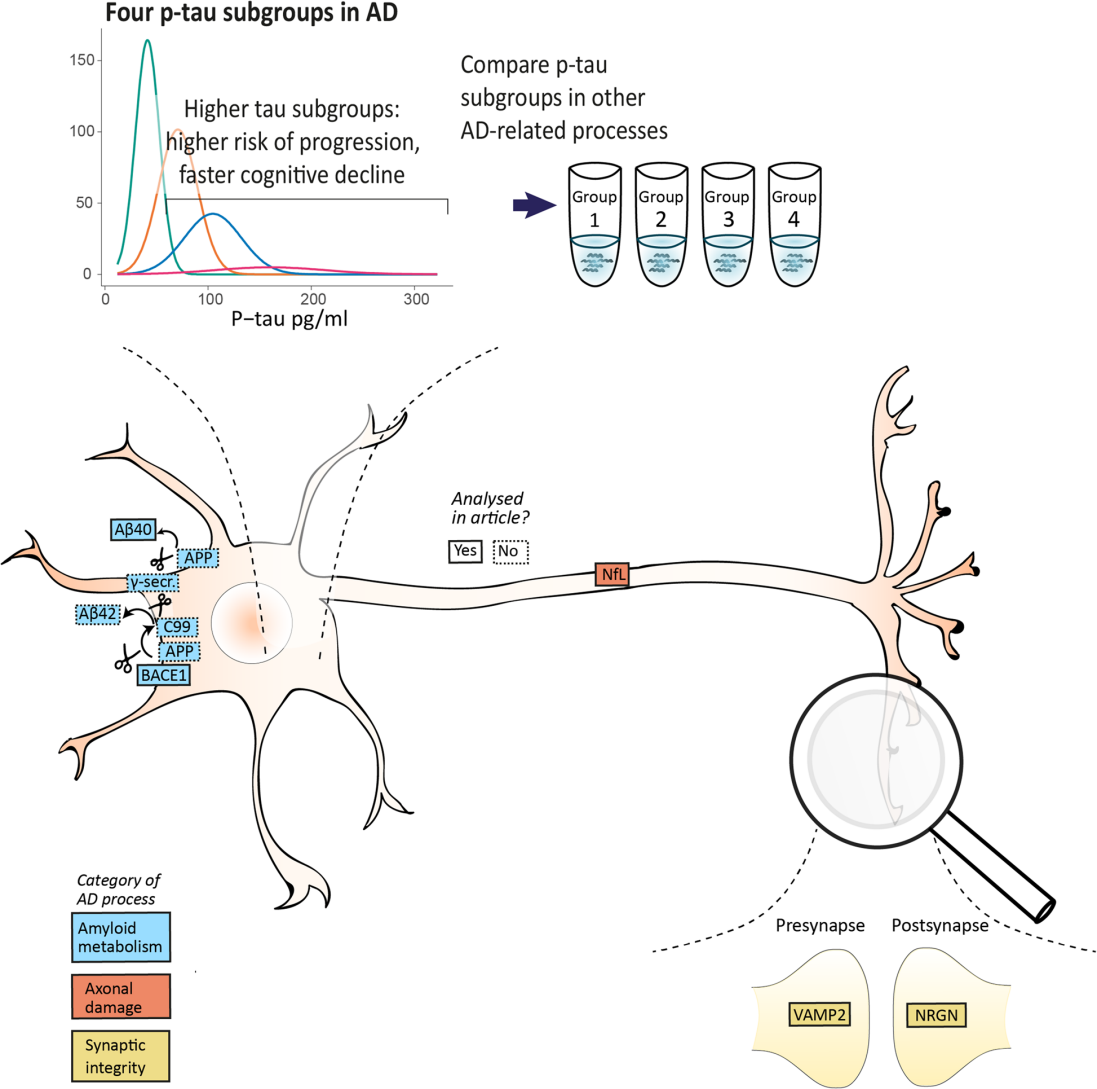
Overview of the study design. Among amyloid abnormal individuals, previously established p-tau subgroups [4] were compared in markers reflecting other AD-related processes: Aβ40 and BACE1 (reflecting amyloid metabolism), NfL (reflecting axonal damage), and VAMP2 and NRGN (reflecting synaptic integrity). The density distribution of p-tau subgroups was adapted from previous research [4]. Aβ40, amyloid-beta 1–40; APP, amyloid precursor protein; BACE1, beta-site amyloid precursor protein cleaving enzyme 1; γ-secr., γ-secretase; NfL, neurofilament light; NRGN, neurogranin; VAMP2, vesicle-associated membrane protein 2

**Fig. 2:**
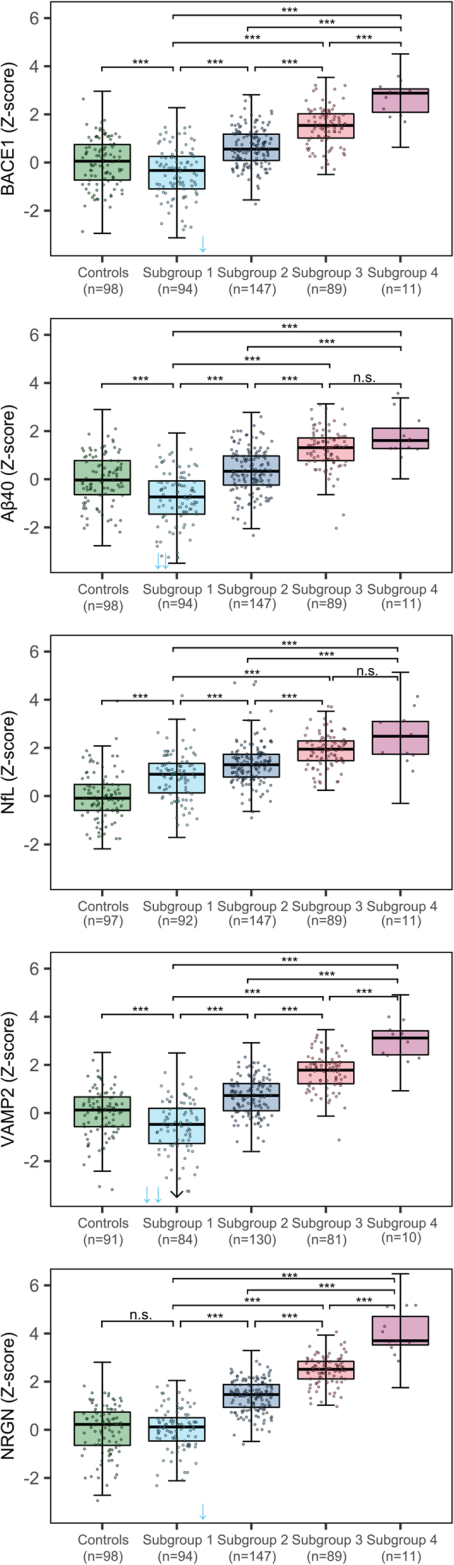
P-tau subgroups differ in proteins reflecting amyloid production, synaptic damage, and axonal damage. Controls were cognitively normal individuals with normal CSF amyloid, t-tau, and p-tau. Protein levels were *Z*-scored relative to controls. The box of the boxplot indicates the 25th percentile, median, and 75th percentile, and whiskers indicate the 1.5× interquartile range. Arrows indicate the protein measurements outside of the *y*-axis. Differences in the protein levels between p-tau subgroups, and between p-tau subgroups and controls, were calculated with linear models corrected for age and sex. *p*-values were adjusted for the multiple comparisons between p-tau subgroups with the Sidak method. BACE1, beta-site amyloid precursor protein cleaving enzyme 1; Aβ40, amyloid-beta 1–40; NfL, neurofilament light; VAMP2, vesicle-associated membrane protein 2; NRGN, neurogranin. *p-value < 0.05; **p-value < 0.01; ***p-value < 0.001; n.s., not significant

**Fig. 3:**
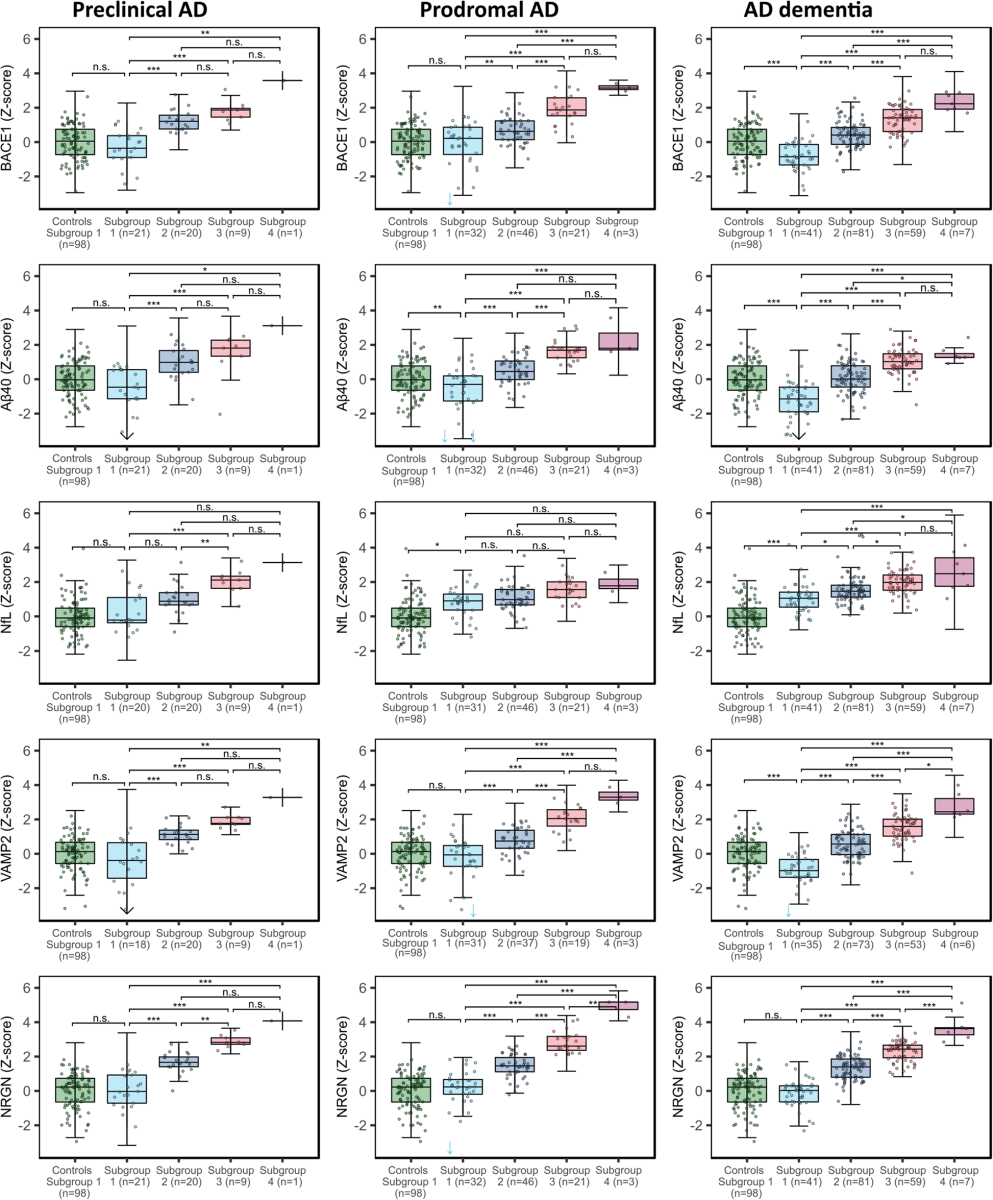
Associations of p-tau subgroups with protein levels were independent of clinical stage. Protein level differences are shown stratified for clinical stage: preclinical AD, prodromal AD, and AD dementia. Controls were cognitively normal individuals with normal CSF amyloid, t-tau, and p-tau. Protein levels were *Z*-scored relative to controls. The box of the boxplot indicates the 25th percentile, median, and 75^th^ percentile, and whiskers indicate the 1.5× interquartile range. Arrows indicate the protein measurements outside of the *y*-axis. Differences between p-tau subgroups in the protein levels were calculated with linear models adjusted for age, sex, diagnostic group (controls, preclinical AD, prodromal AD, and AD dementia), and the interaction between protein level and diagnostic group. *p*-values were adjusted for the multiple comparisons between p-tau subgroups with the Sidak method. BACE1, beta-site amyloid precursor protein cleaving enzyme 1; Aβ40, amyloid-beta 1–40; NfL, neurofilament light; VAMP2, vesicle-associated membrane protein 2; NRGN, neurogranin. *p-value < 0.05; **p-value < 0.01; ***p-value < 0.001; n.s., not significant

Next, we set out to compare the p-tau subgroups in other AD-related processes, including amyloid metabolism, synaptic integrity, and axonal damage (see overview visualization in Fig. 1). Compared to controls, p-tau subgroup 1 showed higher levels of NfL but *lower* levels of BACE1, Aβ40, and VAMP2 (Fig. 2, Table 2). Subgroups 2 to 4 showed stepwise increases in CSF levels of BACE1, VAMP2, NRGN, and Aβ40, of which NRGN showed the largest differences between p-tau subgroups (Fig. 2, Table 2). NfL levels showed stepwise increases across the first three p-tau subgroups and did not reach significance when comparing subgroups 3 and 4. The results remained similar when we repeated the analyses on natural log-transformed and standardized variables and corrected for age and sex effects (Table 3). Repeating analyses including an interaction effect in the models showed that these associations with p-tau subgroups did not differ for disease severity (all interaction *p*-value > 0.1, Table 4, Fig. 3).

**Table 3:** Differences in protein levels between p-tau subgroups, corrected for age and sex

Protein (*Z*-score)	Controls (*n* = 98)	Protein level per p-tau group: mean ± standard error of the mean (SE)				Contrasts between p-tau subgroups													
						Differences between p-tau subgroups: mean ± SE							*p*-values of contrasts						
		Subgroup 1: p-tau ≤ 56 pg/ml (*n* = 94)	Subgroup 2: p-tau 57–96 pg/ml (*n* = 147)	Subgroup 3: p-tau 97–159 pg/ml (*n* = 89)	Subgroup 4: p-tau > 159 pg/ml (*n* = 11)	1 vs control	2 vs 1	3 vs 1	4 vs 1	3 vs 2	4 vs 2	4 vs 3	1 vs control	2 vs 1	3 vs 1	4 vs 1	3 vs 2	4 vs 2	4 vs 3
BACE1 (*n* = 439)	0.11 ± 0.1	-0.46 ± 0.09	0.58 ± 0.07	1.44 ± 0.1	2.58 ± 0.27	− 0.57 ± 0.13	1.05 ± 0.12	1.9 ± 0.13	3.04 ± 0.29	0.85 ± 0.12	1.99 ± 0.28	1.14 ± 0.28	**1.1E−04**	**0.0E+00**	**0.0E+00**	**0.0E+00**	**4.1E−11**	**2.6E−11**	**4.6E−04**
Aβ40 (*n* = 439)	0.18 ± 0.1	-0.82 ± 0.1	0.32 ± 0.08	1.16 ± 0.1	1.67 ± 0.29	− 0.99 ± 0.14	1.14 ± 0.13	1.98 ± 0.15	2.49 ± 0.31	0.84 ± 0.13	1.35 ± 0.3	0.51 ± 0.31	**9.6E−11**	**0.0E+00**	**0.0E+00**	**8.3E−14**	**3.5E−09**	**8.2E−05**	0.513
NfL (*n* = 436)	0.13 ± 0.09	0.76 ± 0.09	1.22 ± 0.07	1.83 ± 0.09	2.36 ± 0.25	0.63 ± 0.12	0.47 ± 0.11	1.07 ± 0.12	1.6 ± 0.26	0.61 ± 0.11	1.14 ± 0.26	0.53 ± 0.26	**2.8E−06**	**1.6E−04**	**7.8E−16**	**1.6E−08**	**4.9E−07**	**8.2E−05**	0.262
VAMP2 (*n* = 396)	0.12 ± 0.1	-0.62 ± 0.1	0.67 ± 0.08	1.64 ± 0.1	2.9 ± 0.29	− 0.74 ± 0.14	1.29 ± 0.13	2.25 ± 0.15	3.52 ± 0.31	0.97 ± 0.13	2.23 ± 0.3	1.27 ± 0.31	**2.3E−06**	**0.0E+00**	**0.0E+00**	**0.0E+00**	**5.7E−12**	**6.6E−12**	**3.1E−04**
NRGN (*n* = 439)	0.1 ± 0.09	0.03 ± 0.08	1.41 ± 0.07	2.43 ± 0.09	3.92 ± 0.24	− 0.07 ± 0.12	1.37 ± 0.11	2.4 ± 0.12	3.89 ± 0.26	1.02 ± 0.11	2.51 ± 0.25	1.49 ± 0.26	0.997	**0.0E+00**	**0.0E+00**	**0.0E+00**	**0.0E+00**	**0.0E+00**	**7.1E−08**

Protein levels were natural log-transformed and standardized relative to cognitively normal individuals with normal CSF amyloid, t-tau, and p-tau (controls). Age- and sex-adjusted differences in protein levels between p-tau subgroups were calculated with linear models. *p*-values of comparisons between p-tau subgroups were adjusted with the Sidak method (considered significant at *p*-value < 0.05)

*CSF*, cerebrospinal fluid; *BACE1*, beta-site amyloid precursor protein cleaving enzyme 1; *Aβ40*, amyloid-beta 1–40; *NfL*, neurofilament light; *NRGN*, neurogranin; *VAMP2*, vesicle-associated membrane protein 2

**Table 4:** Differences in protein levels between p-tau subgroups depending on cognitive stage

Protein (*n*)	Protein level per group: estimate ± SE						Differences between p-tau subgroups: mean ± SE							*p*-values of contrasts						
	Controls (*n* = 98)	Subgroup 1: p-tau ≤ 56 pg/ml (total *n* = 94)	Subgroup 2: p-tau 57–96 pg/ml (total *n* = 147)	Subgroup 3: p-tau 97–159 pg/ml (total *n* = 89)	Subgroup 4: p-tau > 159 pg/ml (total *n* = 11)	Interaction with cognitive stage: *p*-value	1 vs controls	2 vs 1	3 vs 1	4 vs 1	3 vs 2	4 vs 2	4 vs 3	1 vs controls	2 vs 1	3 vs 1	4 vs 1	3 vs 2	4 vs 2	4 vs 3
***Preclinical AD***																				
BACE1 (*n* = 51)	0.1 ± 0.09	− 0.27 ± 0.19	1.23 ± 0.19	1.86 ± 0.29	3.14 ± 0.86	0.381	− 0.37 ± 0.21	1.5 ± 0.27	2.13 ± 0.34	3.41 ± 0.89	0.64 ± 0.34	1.92 ± 0.88	1.28 ± 0.91	0.788	**9.0E−07**	**2.2E−08**	**2.8E−03**	0.755	0.481	0.975
Aβ40 (*n* = 51)	0.17 ± 0.1	− 0.39 ± 0.2	0.96 ± 0.21	1.38 ± 0.31	2.78 ± 0.94	0.831	− 0.55 ± 0.22	1.35 ± 0.29	1.77 ± 0.37	3.17 ± 0.96	0.42 ± 0.37	1.82 ± 0.96	1.4 ± 0.99	0.267	**1.0E−04**	**5.5E−05**	**2.3E−02**	0.998	0.721	0.973
NfL (*n*=50)	0.15 ± 0.09	0.37 ± 0.17	0.92 ± 0.17	2.14 ± 0.26	2.05 ± 0.79	0.123	0.22 ± 0.19	0.56 ± 0.25	1.78 ± 0.31	1.69 ± 0.81	1.22 ± 0.31	1.13 ± 0.8	− 0.09 ± 0.83	0.998	0.412	**5.2E−07**	0.549	**2.3E−03**	0.975	1.000
VAMP2 (*n* = 48)	0.11 ± 0.1	− 0.42 ± 0.21	1.09 ± 0.2	1.91 ± 0.3	2.96 ± 0.91	0.764	− 0.53 ± 0.23	1.51 ± 0.29	2.32 ± 0.37	3.38 ± 0.93	0.81 ± 0.36	1.87 ± 0.93	1.06 ± 0.96	0.383	**8.0E−06**	**1.5E−08**	**7.0E−03**	0.414	0.620	0.999
NRGN (*n* = 51)	0.09 ± 0.09	0.09 ± 0.17	1.65 ± 0.18	2.86 ± 0.26	3.96 ± 0.79	0.403	0 ± 0.19	1.56 ± 0.25	2.77 ± 0.31	3.87 ± 0.81	1.21 ± 0.32	2.31 ± 0.81	1.1 ± 0.84	1.000	**1.3E−08**	**0.0E+00**	**5.7E−05**	**3.0E−03**	0.094	0.988
***Prodromal AD***																				
BACE1 (*n* = 102)	0.1 ± 0.09	− 0.14 ± 0.15	0.62 ± 0.13	1.73 ± 0.19	3.12 ± 0.5	0.381	− 0.24 ± 0.18	0.76 ± 0.2	1.87 ± 0.24	3.26 ± 0.52	1.11 ± 0.23	2.5 ± 0.51	1.38 ± 0.53	0.980	**2.9E−03**	**2.5E−12**	**2.1E−08**	**3.0E−05**	**3.4E−05**	0.180
Aβ40 (*n* = 102)	0.17 ± 0.1	− 0.58 ± 0.17	0.52 ± 0.14	1.55 ± 0.21	2.12 ± 0.54	0.831	− 0.75 ± 0.19	1.11 ± 0.21	2.13 ± 0.27	2.71 ± 0.57	1.03 ± 0.25	1.6 ± 0.56	0.57 ± 0.58	**2.5E−03**	**8.2E−06**	**2.1E−13**	**5.1E−05**	**8.7E−04**	0.088	1.000
NfL (*n* = 101)	0.15 ± 0.09	0.65 ± 0.14	0.84 ± 0.12	1.28 ± 0.17	2.01 ± 0.45	0.123	0.51 ± 0.16	0.18 ± 0.18	0.63 ± 0.22	1.35 ± 0.47	0.44 ± 0.21	1.17 ± 0.47	0.73 ± 0.48	**4.4E−02**	1.000	0.102	0.091	0.506	0.235	0.950
VAMP2 (*n* = 90)	0.11 ± 0.1	− 0.35 ± 0.16	0.69 ± 0.15	1.91 ± 0.21	3.27 ± 0.52	0.764	− 0.46 ± 0.19	1.05 ± 0.22	2.26 ± 0.27	3.62 ± 0.55	1.22 ± 0.26	2.57 ± 0.54	1.36 ± 0.56	0.278	**5.7E−05**	**9.3E−15**	**2.8E−09**	**5.3E−05**	**6.3E−05**	0.287
NRGN (*n* = 102)	0.09 ± 0.09	0.19 ± 0.14	1.45 ± 0.12	2.75 ± 0.18	4.74 ± 0.46	0.403	0.1 ± 0.16	1.26 ± 0.18	2.56 ± 0.22	4.55 ± 0.48	1.3 ± 0.21	3.29 ± 0.47	1.99 ± 0.49	1.000	**3.0E−10**	**0.0E+00**	**0.0E+00**	**2.5E−08**	**2.6E−10**	**1.1E−03**
***AD dementia***																				
BACE1 (*n* = 188)	0.1 ± 0.09	− 0.81 ± 0.13	0.41 ± 0.1	1.27 ± 0.11	2.27 ± 0.32	0.381	− 0.91 ± 0.16	1.22 ± 0.16	2.08 ± 0.18	3.08 ± 0.35	0.86 ± 0.15	1.86 ± 0.34	1 ± 0.34	**6.9E−07**	**1.1E−11**	**0.0E+00**	**0.0E+00**	**2.5E−07**	**1.4E−06**	0.074
Aβ40 (*n* = 188)	0.17 ± 0.1	− 1.22 ± 0.15	0.06 ± 0.1	0.99 ± 0.12	1.33 ± 0.35	0.831	− 1.38 ± 0.18	1.28 ± 0.18	2.21 ± 0.19	2.55 ± 0.38	0.93 ± 0.16	1.27 ± 0.37	0.34 ± 0.37	**7.2E−13**	**6.6E−11**	**0.0E+00**	**1.7E−09**	**2.6E−07**	**1.3E−02**	1.000
NfL (*n* = 188)	0.15 ± 0.09	1.02 ± 0.12	1.5 ± 0.09	1.97 ± 0.1	2.54 ± 0.29	0.123	0.87 ± 0.15	0.48 ± 0.15	0.95 ± 0.16	1.52 ± 0.32	0.47 ± 0.13	1.04 ± 0.31	0.57 ± 0.31	**1.4E−07**	**2.9E−02**	**1.2E−07**	**5.6E−05**	**1.1E−02**	**1.6E−02**	0.762
VAMP2 (*n* = 167)	0.11 ± 0.1	− 0.95 ± 0.15	0.55 ± 0.11	1.5 ± 0.12	2.71 ± 0.37	0.764	− 1.07 ± 0.18	1.5 ± 0.18	2.45 ± 0.2	3.67 ± 0.4	0.95 ± 0.16	2.17 ± 0.38	1.22 ± 0.39	**2.0E−07**	**1.4E−13**	**0.0E+00**	**0.0E+00**	**2.8E−07**	**6.9E−07**	**3.7E−02**
NRGN (*n* = 188)	0.09 ± 0.09	− 0.13 ± 0.12	1.32 ± 0.09	2.25 ± 0.1	3.57 ± 0.3	0.403	− 0.22 ± 0.15	1.45 ± 0.15	2.38 ± 0.16	3.69 ± 0.32	0.93 ± 0.14	2.24 ± 0.31	1.32 ± 0.31	0.961	**0.0E+00**	**0.0E+00**	**0.0E+00**	**5.4E−10**	**4.9E−11**	**7.1E−04**

Protein levels were natural log-transformed and standardized relative to cognitively normal individuals with normal CSF amyloid, t-tau, and p-tau (controls). Estimates of protein level differences between p-tau subgroups are corrected for age and sex and were calculated with linear models which included an interaction term with clinical diagnostic group. *p*-values of comparisons between p-tau subgroups, and between p-tau subgroup 1 and controls, were adjusted with the Sidak method (considered significant at *p*-value < 0.05)

*CSF*, cerebrospinal fluid; *BACE1*, beta-site amyloid precursor protein cleaving enzyme 1; *Aβ40*, amyloid-beta 1–40; *NfL*, neurofilament light; *NRGN*, neurogranin; *VAMP2*, vesicle-associated membrane protein 2

### Associations of p-tau subgroups with protein levels in CN amyloid-negative individuals

**Fig. 4:**
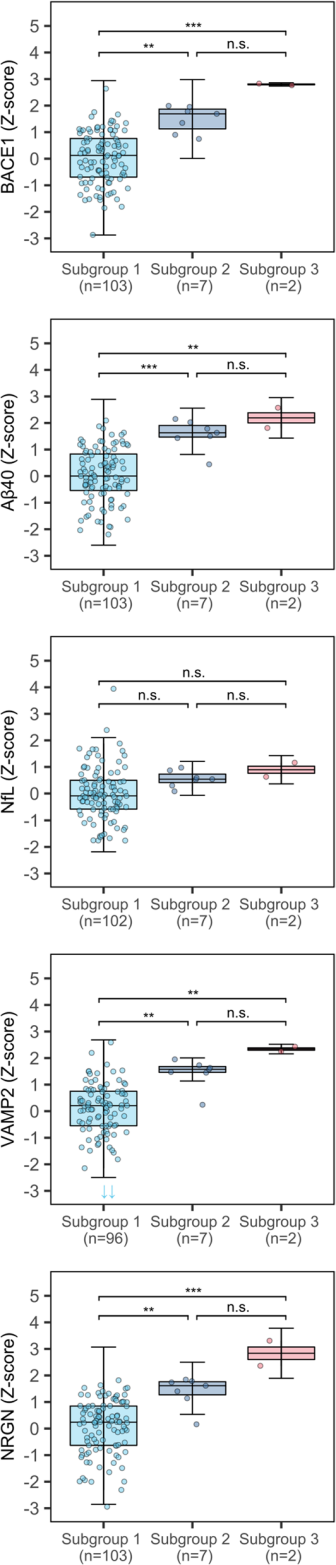
Associations of p-tau subgroups with protein levels in CN amyloid-negative individuals. CN amyloid-negative individuals included both individuals with normal t-tau and p-tau (controls) and individuals with abnormal t- or p-tau (suspected non-Alzheimer’s disease pathology or SNAP). Protein levels were *Z*-scored relative to controls. The box of the boxplot indicates the 25th percentile, median, and 75th percentile, and whiskers indicate the 1.5× interquartile range. Arrows indicate the protein measurements outside of the *y*-axis. Differences between p-tau subgroups in the protein levels were calculated with linear models adjusted for age and sex. *p*-values were adjusted for the multiple comparisons between p-tau subgroups with the Sidak method. BACE1, beta-site amyloid precursor protein cleaving enzyme 1; Aβ40, amyloid-beta 1–40; NfL, neurofilament light; VAMP2, vesicle-associated membrane protein 2; NRGN, neurogranin. *p-value < 0.05; **p-value < 0.01; ***p-value < 0.001; n.s., not significant

Finally, we explored if CN amyloid-negative individuals also show the pattern of increased markers of amyloid production and synaptic damage with higher p-tau subgroups. A minority of these individuals fell in the second p-tau subgroup (*n* = 7/112, 6%) or third p-tau subgroup (*n* = 2/112, 2%). As amyloid-negative individuals with higher p-tau may be in a pre-amyloid stage, we tested if Aβ42 levels differed between p-tau subgroups, but found no differences (Table 5). Levels of BACE1, Aβ40, VAMP2, and NRGN were increased in p-tau subgroup 2 and 3 relative to subgroup 1, while NfL did not differ between the p-tau subgroups (Table 5, Fig. 4).

**Table 5:** Differences in protein levels between p-tau subgroups in amyloid-negative CN individuals

Protein (*n*)	Protein level per group: estimate ± standard error of the mean (SE)			Differences between p-tau subgroups: mean ± SE			*p*-values of contrasts		
	Subgroup 1: p-tau ≤ 56 pg/ml (*n* = 103)	Subgroup 2: p-tau 57–96 pg/ml (*n* = 7)	Subgroup 3: p-tau 97–159 pg/ml (*n* = 2)	2 vs 1	3 vs 1	3 vs 2	2 vs 1	3 vs 1	3 vs 2
*Core AD biomarker*									
Aβ42 (*n* = 112)	0.04 ± 0.11	0.61 ± 0.39	− 0.01 ± 0.74	0.56 ± 0.41	− 0.05 ± 0.74	− 0.62 ± 0.83	0.427	1.000	0.843
*Analytes*									
BACE1 (*n* = 112)	0.03 ± 0.1	1.44 ± 0.37	2.71 ± 0.69	1.4 ± 0.38	2.68 ± 0.69	1.28 ± 0.78	**1.02E−03**	**5.61E−04**	0.276
Aβ40 (*n* = 112)	0.1 ± 0.1	1.55 ± 0.36	2.3 ± 0.69	1.45 ± 0.38	2.2 ± 0.69	0.75 ± 0.77	**6.24E−04**	**5.54E−03**	0.701
NfL (*n* = 111)	− 0.07 ± 0.09	0.36 ± 0.31	0.34 ± 0.59	0.42 ± 0.32	0.41 ± 0.59	− 0.02 ± 0.66	0.470	0.869	1.000
VAMP2 (*n* = 105)	0.07 ± 0.11	1.4 ± 0.38	2.3 ± 0.72	1.33 ± 0.39	2.23 ± 0.72	0.9 ± 0.8	**3.16E−03**	**7.51E−03**	0.604
NRGN (*n* = 112)	0.09 ± 0.1	1.37 ± 0.38	2.88 ± 0.71	1.29 ± 0.39	2.79 ± 0.72	1.5 ± 0.8	**4.25E−03**	**5.29E−04**	0.180

Protein levels were natural log-transformed and standardized relative to cognitively normal individuals with normal CSF amyloid, t-tau, and p-tau (controls). Estimates of protein level differences between p-tau subgroups are corrected for age and sex. *p*-values of comparisons between p-tau subgroups were adjusted with the Sidak method (considered significant at *p*-value < 0.05)

*CN*, cognitively normal; *CSF*, cerebrospinal fluid; *BACE1*, beta-site amyloid precursor protein cleaving enzyme 1; *Aβ42*, amyloid-beta 1–42; *Aβ40*, amyloid-beta 1–40; *NfL*, neurofilament light; *NRGN*, neurogranin; *VAMP2*, vesicle-associated membrane protein 2

## Discussion

We found that among individuals with AD, the lowest p-tau subgroup showed reduced levels of amyloid production biomarkers (BACE1 and Aβ40) and a presynaptic marker (VAMP2) relative to controls. Subgroups with gradually higher p-tau values (subgroups 2–4) showed stepwise increases in BACE1, Aβ40, VAMP2, a postsynaptic biomarker (NRGN), and, to a more moderate extent, an axonal damage marker (NfL). As these associations were independent of clinical stage, and were similar for amyloid-negative CN individuals (apart from NfL which did not show differences in this group), p-tau subgroups may, in addition to disease severity, reflect biological subtypes of AD that have distinct profiles of amyloid metabolism and synaptic integrity markers, apparently already when cognition is still unimpaired.

We found that p-tau subgroups 2–4 had increasingly higher CSF markers of amyloid production and synaptic integrity markers, which is in line with studies that reported continuous associations of tau with biomarkers reflecting these processes (i.e., Aβ40 [36] and BACE1 [12, 13], NRGN [8, 18–22], and VAMP2 [16]). A previous study also showed tau and amyloid metabolism may have overlapping causal mechanisms, as in cognitively intact monozygotic twins, amyloid production markers in one twin were related to p-tau levels of the other twin [37]. A similar result was observed in APPPS1 mice, which do not develop tau pathology but nonetheless showed increasing CSF p-tau levels over time which closely corresponded to increasing amyloid load [38]. Our results provide further support for the idea that processes of tau and amyloid metabolism may be linked. Since in normal cognition high tangle burden is not expected, this suggests that p-tau levels in CSF may reflect other pathophysiological aspects of AD as well. Subgroup 1 consisted mostly of individuals with normal CSF p-tau, and also included individuals with dementia, which are thought to have the highest pathological tau burden. In the current research framework, AD is defined based on abnormal amyloid and tau biomarkers [2]. This framework suggests that the subgroup with normal p-tau CSF levels and abnormal amyloid (i.e., A+T−) do not have AD, but AD pathological change. However, previous studies found that up to 30% of individuals with a pathological diagnosis of AD, can show normal CSF p-tau levels [39]. Possibly, normal p-tau levels may indicate a biological subtype of AD, since individuals in this subgroup showed compared to controls a different pattern of alterations in CSF markers. For example, VAMP2 and amyloid metabolism markers were decreased compared to controls, whereas these markers were increased in subgroups 2–4. This was not a generic effect, because NfL was increased in subgroup 1 compared to controls. This suggests that this tau subgroup shows a distinct underlying pathophysiology that is related to lower p-tau levels in CSF. A recent CSF proteomic study suggests that A+ individuals with normal tau levels show involvement in blood-brain barrier dysfunction, and indications of decreased amyloid metabolism as well as lower levels of proteins associated with neuronal plasticity [39]. Potentially, the lowest tau subgroup could differ in additional processes, e.g., in another study, A+ individuals with normal tau levels showed reductions in immune-related proteins [40]. Together, our results provide further evidence that the four subgroups we observed in CSF p-tau levels reflect, at least in part, distinct pathophysiological processes. Further studies should aim to investigate these processes in more detail, e.g., through a combined CSF and pathology approach.

Compared to the other markers, we observed that the increases in NfL levels with higher p-tau subgroups were less pronounced and did not differ between the third and fourth p-tau subgroup. Potentially, associations of p-tau subgroups with amyloid and synaptic markers may be stronger compared to NfL, because structural axonal loss occurs downstream of changes in amyloid production and synaptic damage [22, 41, 42]. Overall, our results suggest focusing on amyloid and synaptic processes rather than axonal damage may be promising to further characterize p-tau subgroups.

Finally, we observed that levels of amyloid production markers were increased with higher p-tau subgroups even in amyloid-negative CN individuals. A previous study showed that higher levels of markers for amyloid production were associated with a steeper (i.e., more abnormal) decline in Aβ42 levels [43]. Possibly, such high amyloid metabolism markers in combination with high tau levels may represent a very early stage of AD, and future studies should aim to test this hypothesis in a longitudinal design with repeated CSF sampling.

There is currently one drug candidate in a phase III clinical trial that targets tau aggregation (TRx0237, ClinicalTrials.gov identifier NCT03446001), and several additional drug candidates targeting tau are currently undergoing phase I and II trials [44, 45]. Our p-tau cutoffs could be useful to select individuals with high p-tau levels as participants for clinical trials of drug candidates targeting tau, who may hypothetically benefit most from lowering their p-tau levels. Additionally, including amyloid production and synaptic markers as exploratory trial outcomes could be insightful to show if reductions in p-tau will also normalize markers of these processes.

## Limitations

A limitation of this study was that we analyzed the p-tau subgroups cross-sectionally. Although we previously showed in ADNI that the majority of individuals remain in their p-tau subgroup over time [4], it remains to be determined if levels of the other proteins remain similarly constant. Another potential limitation of the present study is that we selected 5 proteins a priori. More proteins reflecting other biological processes may be involved that could be different as well, and future studies should investigate this using, e.g., proteomic approaches. Subgroup 4 had a relatively small sample size, and follow-up studies in even larger sample sizes will be useful to replicate the findings. Nonetheless, a strength of this study was that we had an overall large sample size of 453 individuals across the AD clinical spectrum and CN amyloid-negative individuals from a well-defined cohort, from whom CSF was collected under standardized biobanking conditions. Additionally, to our knowledge, this is the first study with a large sample size to validate that VAMP2 is associated with AD.

## Conclusions

In conclusion, we found that subgroups of individuals with increasingly high p-tau showed stepwise increases in proteins reflecting amyloid production and synaptic damage. Our data suggest that heterogeneity in tau pathology is related to differences in amyloid production and synaptic processes, which seems independent of the clinical stage. P-tau subgroups might be useful as a stratification tool to select individuals with AD, including those still in preclinical and prodromal stages, as participants for clinical trials targeting tau aggregation.

## Data Availability

The dataset supporting the conclusions of this article is available from the corresponding author upon reasonable request.

## Abbreviations

A+: Amyloid abnormal
Aβ40: Amyloid-beta 1–40
Aβ42: Amyloid-beta 1–42
AD: Alzheimer’s disease
ADC: Amsterdam Dementia Cohort
*APOE*: Apolipoprotein E
APP: Amyloid precursor protein
BACE1: Beta-site amyloid precursor protein cleaving enzyme 1
CN: Cognitively normal
CSF: Cerebrospinal fluid
CV: Coefficient of variation
EEG: Electroencephalography
FDR: False discovery rate
MCI: Mild cognitive impairment
MMSE: Mini-Mental State Examination
MRI: Magnetic resonance imaging
NfL: Neurofilament light
NIA-AA: National Institute on Aging and Alzheimer’s Association
NINCDS-ADRDA: National Institute of Neurological and Communicative Disorders and Stroke-Alzheimer’s Disease and Related Disorders Association
NRGN: Neurogranin
p-tau: Tau phosphorylated at threonine 181
SIMOA: Single-molecule array
SNAP: Suspected non-Alzheimer’s disease pathophysiology
t-tau: Total tau
VAMP2: Vesicle-associated membrane protein 2
